# When crisis awareness weakens rational evaluation: the conditional role of information quality in college English teachers' adoption of generative AI

**DOI:** 10.3389/fpsyg.2026.1822948

**Published:** 2026-04-28

**Authors:** Yan Cheng, Haibo Liu

**Affiliations:** 1 School of General Education, Shandong Huayu University of Technology, Dezhou, China; 2 College of Mathematics and Computer, Jilin Normal University, Siping, China; 3 Dezhou Information Engineering Secondary Vocational School, Dezhou, China

**Keywords:** adoption intention, crisis awareness, generative artificial intelligence, information quality, technology acceptance model, Threat Rigidity Theory

## Abstract

Generative artificial intelligence (GenAI) is rapidly transforming higher education. However, the psychological mechanisms underlying its adoption among educators remain insufficiently understood. Drawing on an extended Technology Acceptance Model (TAM) and Threat Rigidity Theory (TRT), this study examines how cognitive evaluations and crisis awareness (CA) jointly influence college English teachers' willingness to adopt (WTA) GenAI. Data were collected from 335 university English teachers in China and analyzed using structural equation modeling. The results show that perceived usefulness and information quality are significant predictors of WTA, whereas perceived ease of use and CA do not exert significant direct effects. More importantly, CA exhibits a conditional moderating role. It significantly weakens the positive relationship between information quality and WTA. This finding suggests that under high levels of perceived professional threat, teachers may rely less on rational evaluation of AI-generated content and instead prioritize rapid adaptation. This study extends the TAM by integrating informational and psychological factors and reveals the selective boundary conditions of CA in artificial intelligence adoption.

## Introduction

Generative artificial intelligence (GenAI) has emerged as a disruptive technological paradigm in higher education (Zhang and Shi, 2025). This technology significantly transforms traditional pedagogical models. Unlike passive multimedia tools, GenAI systems exhibit profound agency, generativity, and autonomy (Li et al., 2025; Liu et al., 2024). They can autonomously perform complex tasks, such as generating instructional content, providing personalized feedback, and evaluating linguistic nuances. Consequently, they redefine the boundaries of educational technology (Cambra-Fierro et al., 2025; Castaneda and Selwyn, 2018; Huang C. et al., 2026). For college English teachers, GenAI presents unprecedented opportunities to enhance curriculum design and alleviate repetitive grading burdens (Qu et al., 2024; Zhang et al., 2026).

However, the adoption of GenAI among educators is neither automatic nor purely rational. The transition from traditional interaction between humans and computers to collaboration between humans and artificial intelligence introduces profound cognitive complexities. Teachers' willingness to adopt (WTA) new technologies is a multifaceted process. It is influenced by cognitive evaluations, affective responses, and psychological resistance (Bailey et al., 2022; Chen X. J. et al., 2025; Foroughi et al., 2024). The traditional Technology Acceptance Model (TAM) provides a foundational framework (Davis, 1989). Yet, it predominantly focuses on the instrumental utility of technology. GenAI capabilities heavily overlap with the core professional competencies of language teachers, including translation and writing instruction. This integration triggers unique psychological barriers. Common issues include algorithm trust deficits, professional identity crises, and anxiety over generative abuse. The traditional TAM framework does not fully explain these issues (Liu and Mao, 2026; Zuo et al., 2025).

Specifically, the psychological state of crisis awareness (CA) represents a critical factor. This study defines CA as the perceived professional threat and urgency arising from rapid AI advancements (Hu et al., 2022; Zuo et al., 2025). General technology acceptance models assume a rational evaluation process. Nevertheless, the presence of CA introduces potential psychological deviations. According to Threat Rigidity Theory (TRT), individuals under perceived threat tend to think more narrowly and rely on simple rules rather than careful analysis (Staw et al., 1981; Grassini, 2023). Based on this premise, we postulate that excessive CA might trigger defensive responses. Teachers may adopt AI quickly to cope with pressure, rather than carefully evaluating it (Chen H. et al., 2025; Cho et al., 2025). The core utility of GenAI relies on its generative outputs. Therefore, we deduce that high CA levels might undermine the rational evaluation of specific system attributes, particularly Information Quality (IQ).

Empirical evidence regarding how CA conditionally alters rational adoption pathways remains scarce. This gap raises a critical research question. How do technical attributes like IQ interact with psychological stressors like CA to shape GenAI adoption? Furthermore, under what boundary conditions does psychological pressure interfere with rational technology evaluation? To address these questions, the present study has two main objectives. First, it extends the TAM framework by integrating IQ and CA to reflect the generative uncertainty of GenAI. Second, it examines the moderating effect of CA on the relationships between traditional TAM variables and WTA based on the theory of threat rigidity.

This study makes two main contributions. Theoretically, it situates GenAI adoption within a novel cognitive and psychological framework. This approach extends the boundaries of TAM in the AI era. It also explores the potential dual role of CA as both a driver and a constraint (Choung et al., 2023; Jiao and Cao, 2024). Practically, the findings offer actionable guidance for university administrators. The study encourages a shift from merely mandating technology use to actively managing psychological thresholds. This shift is essential for fostering a supportive ecosystem and achieving sustainable symbiosis between humans and AI in higher education (Chen and Gu, 2025; Dorji and Nima, 2025; Lau et al., 2025).

## Literature review and research hypotheses

### Theoretical foundation: Threat Rigidity Theory

TRT provides a fundamental psychological framework for understanding how individuals respond to external pressures. Originally conceptualized by Staw et al. (1981), the theory posits that individuals facing severe external threats exhibit fundamentally altered decision-making processes. When exposed to high-stress situations, individuals typically experience two primary psychological manifestations: cognitive narrowing and a reliance on rigid heuristics (Schittko et al., 2025; Grassini, 2023; Staw et al., 1981).

Cognitive narrowing refers to a significant restriction in information processing capacity. Under acute threat, individuals suffer from cognitive overload and emotional distress. Consequently, they lose the cognitive flexibility required to absorb, evaluate, and integrate complex new information. They become hyper-focused on immediate survival strategies or escaping the source of anxiety (Chen et al., 2021b; Pearsall et al., 2009). Simultaneously, this cognitive depletion forces individuals to rely on established routines, superficial cues, or compliance-driven behaviors rather than engaging in rational, analytical thinking (Cho et al., 2025).

In the context of this study, TRT serves as a crucial theoretical lens. The rapid integration of GenAI in higher education presents a profound professional threat to college English teachers. While traditional technology acceptance models assume a rational evaluation of system attributes, TRT suggests that intense psychological pressure disrupts this rationality. When teachers experience high CA, their cognitive resources are diverted toward managing anxiety and identity defense. This defensive state likely undermines their ability to objectively evaluate complex technical features, thereby shifting from careful adoption to more passive use under pressure.

### TAM in the era of GenAI

The TAM, originally proposed by Davis (1989), remains one of the most robust theoretical frameworks for predicting users' intentions to adopt new information systems. The core premise of TAM states that an individual's behavioral intention to use a technology is determined by two cognitive beliefs. Perceived Usefulness (PU) refers to the degree to which individuals believe that using a particular system enhances their job performance. Perceived Ease of Use (PEU) denotes the extent to which they perceive using the system to be free of effort. Over the decades, TAM has been successfully extended to various contexts. These include digital learning, smart campus technologies, and digital service platforms (Bali et al., 2024; Cesme and Cimen, 2026; Lisha et al., 2025; Puiu and Udristioiu, 2024; Yang and Qian, 2025; Seyum et al., 2025; Zhang and Jing, 2026).

The advent of GenAI has prompted scholars to reexamine the validity of TAM. A recent comprehensive review by Xue et al. (2026) confirmed that the foundational TAM relationships remain highly significant in the context of artificial intelligence in education (Choi et al., 2022; Zuo et al., 2022). They often exhibit stronger predictive power than in traditional online learning domains. However, GenAI introduces a fundamental paradigm shift. Traditional educational tools are deterministic in nature with fixed and highly predictable outputs. By contrast, GenAI functions as a collaborative agent capable of generating creative problem-solving solutions. This capability fundamentally alters the nature of users' interactions with technological systems (Han and Tepsan, 2026; Li and Wagner, 2026; Sogemeier et al., 2025).

In the specific context of college English teaching, PU reflects how effectively GenAI can assist educators in advanced pedagogical tasks. These tasks involve tailoring reading materials to varying proficiency levels, simulating intercultural dialogues, and accelerating the grading of complex essays (Lee and Tseng, 2025; Leiss et al., 2025). PEU, meanwhile, has shifted from traditional software navigation to the mastery of prompt engineering. This skill involves the ability to effectively communicate with large language models to elicit desired outputs (Grace et al., 2025; Huang X. et al., 2026; Liew et al., 2025). When teachers perceive GenAI as highly capable of improving teaching efficiency and relatively easy to interact with, their intrinsic motivation to integrate these tools increases significantly. Aligning with the extended applications of TAM in recent GenAI studies, we hypothesize:

H1: PU positively influences college English teachers' willingness to adopt GenAI.
H2: PEU positively influences college English teachers' willingness to adopt GenAI.

### Information quality and generative uncertainty

IQ refers to the user's perception of the accuracy, completeness, reliability, timeliness, and contextual relevance of system outputs. While IQ has long been recognized as a critical factor in information system success, its theoretical weight has increased exponentially in the GenAI era. This increase is primarily due to the phenomenon of generative uncertainty (Bryant et al., 2026; Wang et al., 2013). Unlike traditional databases that retrieve preexisting verified information, LLMs generate probabilistic outputs based on statistical patterns. This mechanism makes GenAI susceptible to AI hallucinations, algorithmic bias, and a lack of specific pedagogical context (Serrano, 2026).

For foreign language teachers, the cost of inaccurate information is exceptionally high. Misleading linguistic feedback, incorrect grammatical explanations, or culturally inappropriate translations generated by AI can directly compromise instructional integrity. Such errors ultimately harm student learning outcomes (Zhang et al., 2026). Recent empirical evidence emphasizes that users' trust in AI-generated content dictates their continuous usage intention. For instance, Marjerison et al. (2025) and Gibbard et al. (2026) demonstrated that the perceived functional value of AI tools is strictly contingent on the informational reliability and transparency of the outputs. If the generated content requires extensive factual verification and manual correction by the teacher, the perceived utility of the tool diminishes entirely. Conversely, if teachers perceive GenAI outputs as highly accurate, pedagogically sound, and immediately applicable to their teaching context, their willingness to adopt increases. Thus, we hypothesize:

H3: IQ positively influences college English teachers' willingness to adopt GenAI.

### Crisis awareness and defensive motivation

Crisis Awareness (CA) in this study refers to teachers' perceived sense of urgency, competitive pressure, and anxiety about falling behind in response to the rapid advancement of GenAI. In the context of higher education, foreign language teachers are particularly vulnerable to such pressures. The core functionalities of large language models closely overlap with traditional instructional tasks (Zuo et al., 2025). As a result, educators may experience heightened concerns about maintaining professional competitiveness and adapting to evolving technological environments (Hu et al., 2022).

The influence of CA on technology adoption can be understood through Cognitive Appraisal Theory (Folkman, 2020). This theory suggests that individuals interpret environmental stressors either as challenges or as threats. When perceived as a challenge, CA functions as a motivational driver, encouraging teachers to actively engage with emerging technologies to maintain professional competitiveness (Lin et al., 2025; Xia et al., 2026). In this sense, moderate levels of CA may stimulate proactive learning, experimentation, and technology adoption.

However, when CA becomes excessive, it operates as a hindrance stressor. High levels of perceived threat can trigger defensive psychological responses, including anxiety, cognitive overload, and identity-related concerns. According to TRT, individuals experiencing high external threat tend to exhibit cognitive narrowing and reduced information processing flexibility (Staw et al., 1981; Grassini, 2023). Under such conditions, teachers may shift from reflective assessment to more immediate coping strategies, potentially resulting in superficial or compliance-driven adoption behaviors (Chen H. et al., 2025; Cho et al., 2025).

Taken together, these perspectives suggest that CA may activate competing psychological mechanisms. While it can motivate proactive engagement under moderate levels, it may also inhibit rational evaluation under high levels of perceived threat. Given that CA may function as both a motivating driver and a hindering stressor, its net effect on adoption intention remains theoretically ambiguous. Therefore, rather than assuming a predetermined directional effect, this study proposes that CA is significantly associated with teachers' willingness to adopt generative AI.

H4: Crisis awareness (CA) is significantly associated with college English teachers' willingness to adopt (WTA) GenAI.

### The moderating role of crisis awareness

While the direct relationships between perceived technological characteristics and adoption intention are well-established within TAM, translating these cognitive evaluations into actual behavioral intention is highly contingent upon the psychological state of the user. In higher education, the rapid proliferation of GenAI has introduced a significant degree of professional vulnerability. CA represents the psychological pressure and sense of professional displacement experienced by educators when confronted with disruptive technologies. We argue that CA acts as a crucial boundary condition. It moderates the structural relationships between PU, PEU, IQ, and WTA.

Integrating TRT into the context of GenAI adoption reveals a complex interaction effect regarding how educators process technology-related stress (Staw et al., 1981; Grassini, 2023). When college English teachers experience intense CA triggered by rapid technological advancements, their focus shifts from evaluating usefulness to managing their emotions and protecting their professional role (Liu et al., 2026). According to the core tenets of this psychological mechanism, high levels of psychological distress and perceived external threats severely restrict information processing capabilities. They also diminish overall cognitive flexibility (Staw et al., 1981).

In modern educational settings, evaluating IQ is a rigorous task. It intrinsically requires a high cognitive load, sustained critical thinking, and objective pedagogical analysis to carefully verify the accuracy, relevance, and safety of AI-generated outputs (Akbar, 2025). Under conditions of high CA, teachers frequently experience cognitive narrowing. In this situation, teachers may pay less attention to carefully evaluating information and instead adopt AI more quickly to cope with pressure. Consequently, highly anxious educators are significantly more likely to blindly adopt GenAI tools merely to alleviate immediate professional threats and avoid falling behind their peers. They become notably less sensitive to the actual quality of the generated information (Chiu et al., 2023; Wang and Wang, 2026). This indicates that the positive driving force of IQ on WTA is heavily compromised when teachers are overwhelmed by a professional crisis.

Conversely, PU and PEU operate as generalized heuristic beliefs within TAM. These beliefs demand relatively less continuous cognitive effort compared to the rigorous verification of IQ (Davis, 1989; Venkatesh and Bala, 2008). While the effects of these foundational perceptions on adoption intention remain significant, the psychological pressure exerted by CA introduces variability into how teachers weigh usefulness and ease of use. Therefore, we expect excessive CA to systematically alter the strength of these structural relationships by weakening the positive effects of rational technological evaluations. Accordingly, we propose the following moderation hypotheses:

H5a: CA negatively moderates the relationship between PU and WTA. Specifically, the positive effect of PU becomes weaker under higher levels of CA.
H5b: CA negatively moderates the relationship between PEU and WTA. Specifically, the positive effect of PEU becomes weaker under higher levels of CA.
H5c: CA negatively moderates the relationship between IQ and WTA. Specifically, the positive effect of IQ becomes weaker under higher levels of CA.

**Figure 1:**
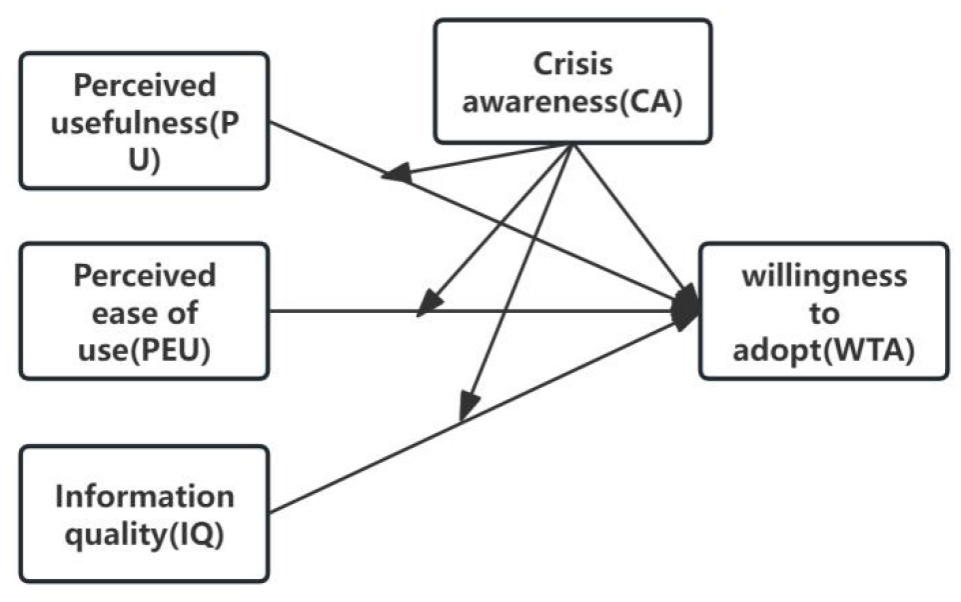
Research model.

Based on the theoretical framework and proposed hypotheses, the conceptual research model is illustrated in Figure 1.

## Methodology

### Sample and data collection

To empirically test the proposed research model, data were collected through an online questionnaire survey administered via Wenjuanxing, a widely used professional survey platform in China. This approach enabled efficient data collection and ensured broad geographical coverage of participants.

Participants were recruited using a purposive sampling strategy targeting college English teachers from multiple higher education institutions across China. To ensure that respondents had relevant experience with GenAI, a screening question was included at the beginning of the questionnaire, asking whether they had used GenAI tools in their teaching or work-related activities. Only those who answered affirmatively were allowed to proceed with the survey.

Prior to participation, all respondents were provided with detailed information about the purpose of the study, and were assured that their responses would remain anonymous and confidential. Participation was entirely voluntary. Electronic informed consent was obtained from all participants before they completed the questionnaire, and they were informed of their right to withdraw from the study at any time without penalty. This study was approved by the Institutional Review Board of Shandong Huayu University of Technology.

Data collection lasted approximately 2 weeks and yielded a total of 364 initial responses. To ensure the quality and reliability of the data, a rigorous screening process was applied. Specifically, responses were excluded if (1) the completion time was < 110 s, indicating insufficient engagement; (2) identical answers were selected across all items (straight-lining); or (3) respondents reported no prior experience with GenAI tools. After data cleaning, 335 valid questionnaires were retained for subsequent analysis, resulting in an effective response rate of 92.03%.

The demographic characteristics of the participants are presented in Table 1. Overall, the sample exhibits a reasonable distribution in terms of gender and GenAI usage experience, providing a reliable basis for subsequent statistical analyses. However, as a non-probability purposive sampling method was adopted, caution should be exercised when generalizing the findings beyond the sample.

**Table 1:** Descriptive statistics of the sample.

Type	Variable	Frequency	Percentage (%)
Gender	Male	122	36.4
	Female	213	63.6
GenAI usage experience	Within 3 months	200	59.7
	3–6 months	62	18.5
	More than 6 months	73	21.8

### Instruments development

In this study, the survey instrument consisted of two main sections. The first section collected participants' demographic information, including gender and GenAI usage experience. The second section comprised measurement items for the five core latent variables in the research model, namely PU, PEU, IQ, CA, and WTA.

All measurement items were adapted from well-validated domestic and international literature to ensure content validity. Minor wording modifications were made to tailor the items to the context of college English teaching and the application of GenAI technologies. The questionnaire was originally developed in English and translated into Chinese using a standard translation procedure. To ensure linguistic accuracy and contextual appropriateness, a back-translation process was conducted. To further ensure content validity, three experts in applied linguistics and educational technology were invited to review the questionnaire. They evaluated the clarity, relevance, and appropriateness of each item. Based on their feedback, minor revisions were made to improve wording and clarity. Before the formal survey, a pilot test was conducted with a small sample of college English teachers to assess the comprehensibility and feasibility of the instrument. The results indicated that the questionnaire was clear and easy to understand. Several minor adjustments were made accordingly. After data collection, the reliability and validity of the measurement scales were assessed. All constructs were measured using a five-point Likert scale ranging from 1 (strongly disagree) to 5 (strongly agree), as shown in Table 2.

**Table 2:** Measurement scales and items.

Construct	Item code	Measurement items
Perceived usefulness (PU)	PU1	Using GenAI software improves my teaching and work efficiency.
	PU2	GenAI software enriches my teaching content and resources (e.g., checking grammar, generating assignments and exam questions, summarizing documents, and writing texts).
	PU3	Using GenAI software enhances the overall quality of my teaching.
Perceived ease of use (PEU)	PEU1	I find it easy to master the various functions provided by GenAI software.
	PEU2	It is easy for me to access or acquire GenAI software (e.g., via app stores or shared links).
	PEU3	I can easily explain how to operate GenAI software to others.
Information quality (IQ)	IQ1	The information generated by GenAI software meets my needs in terms of completeness.
	IQ2	The information generated by GenAI software meets my needs in terms of accuracy.
	IQ3	The information provided by GenAI software meets my needs in terms of timeliness (e.g., update frequency).
Crisis awareness (CA)	CA1	Given the rapid development of the information society, I fear that not using GenAI software will cause me to fall behind others technologically.
	CA2	I feel that if I do not use GenAI software, I will be unable to access new information rapidly.
	CA3	I worry that if I do not use GenAI software, my efficiency in handling daily work tasks will decrease.
Willingness to adopt (WTA)	WTA1	I am willing to use GenAI software for teaching activities and administrative tasks in the future.
	WTA2	I am willing to recommend GenAI software to my colleagues and students.
	WTA3	I believe that the application of GenAI software is an inevitable trend in the future development of higher education.

#### Perceived usefulness (Cronbach's α = 0.762)

PU was adapted from the classic scale developed by Davis (1989). This construct was measured using three items assessing the extent to which GenAI enhances teaching efficiency and effectiveness. A sample item is: “Using GenAI software improves my teaching and work efficiency.” The scale demonstrated acceptable reliability.

#### Perceived ease of use (Cronbach's α = 0.787)

PEU was adapted from Chen et al. (2015). This construct included three items focusing on the perceived simplicity and ease of operation of GenAI tools. A sample item is: “I find it easy to master the various functions provided by GenAI software.” The scale showed good reliability.

#### Information quality (Cronbach's α = 0.867)

Information quality was derived from Liu et al. (2024). This variable was measured using three items evaluating the accuracy, completeness, and timeliness of information generated by GenAI systems. The scale demonstrated strong reliability.

#### Crisis awareness (Cronbach's α = 0.764)

Crisis awareness was measured using a self-developed three-item scale. In this study, CA is conceptualized as teachers' perceived sense of urgency, competitive pressure, and anxiety about falling behind in response to the rapid advancement of GenAI. Although prior studies have conceptualized CA in terms of professional identity threat and potential job displacement (Bandura and Adams, 1977; Folkman, 2020), the present study operationalizes CA as AI-induced urgency and performance-related pressure. This operationalization reflects more immediate and observable psychological responses in teachers' day-to-day interactions with generative AI technologies. Given the lack of well-established scales tailored to this specific context, the measurement items were developed with reference to prior research on technology-related pressure perception and adaptation (Liu and Mao, 2026; Zuo et al., 2025).

#### Willingness to adopt (Cronbach's α = 0.836)

Willingness to adopt was adapted from Pan et al. (2016). As the dependent variable in this study, it was measured using three items assessing teachers' intentions to use and recommend GenAI tools in their future teaching and work practices. A sample item is: “I am willing to use GenAI software for teaching activities and administrative tasks in the future.” The scale demonstrated strong reliability.

### Data analysis

Data analysis proceeded sequentially using SPSS 27.0 and AMOS 26.0 (Anderson and Gerbing, 1988). First, preliminary assessments evaluated data normality through skewness and kurtosis metrics. Common method bias was rigorously addressed using a tri-method approach to ensure data integrity. This procedure included the traditional Harman single-factor test, confirmatory factor analysis (CFA) model comparisons, and the unmeasured latent method construct (ULMC) technique. Second, the measurement model was validated via CFA. Internal consistency was confirmed through Cronbach alpha and composite reliability coefficients. Convergent validity was established using standardized factor loadings and average variance extracted (AVE). Discriminant validity was strictly verified using the Fornell–Larcker criterion. Third, structural equation modeling (SEM) tested the direct hypotheses (H1–H4). Overall model fit was evaluated using established absolute and incremental fit indices, including χ^2^/df, AGFI, NFI, TLI, CFI, and RMSEA. Finally, conditional hypotheses (H5a–H5c) were tested using the SPSS PROCESS macro. Predictor variables were mean-centered prior to analysis to prevent multicollinearity. Bootstrapping with 5,000 resamples generated 95% confidence intervals to determine the precise statistical significance of the interaction terms (Wang and Wang, 2026).

## Results

### Assessment of normality and common method bias assessment

First, all measurement items were examined for skewness and kurtosis. The results showed that skewness values ranged from −0.757 (±0.133) to −0.042 (±0.133), all well-below the threshold of three. Kurtosis values ranged from −0.108 (±0.266) to 2.22 (±0.266), also below the recommended threshold of 10. These findings indicate that the data generally satisfy the assumption of normal distribution.

Given the self-reported nature of the survey, common method bias (CMB) was initially assessed using Harman's single-factor test. The unrotated exploratory factor analysis showed that the first factor accounted for 21.11% of the total variance, which is below the commonly accepted threshold of 40% (Liu et al., 2026). This suggests that CMB is unlikely to be a serious concern in this study.

To further examine potential CMB, both the CFA comparison approach and the ULMC technique were employed.

For the CFA comparison, a single-factor model was constructed by loading all items onto one latent factor and compared with the proposed five-factor model. As shown in Table 3, the five-factor model exhibited a significantly better fit than the single-factor model (Δχ^2^ = 517.455, Δdf = 10, and *p* < 0.001). This result indicates that the measurement model captures distinct constructs and that CMB is not substantial.

**Table 3:** Changes of CFA comparison model.

Model	χ^2^	df	Δχ^2^	Δdf	*p*
One-factor	687.657	90	517.455	10	< 0.001
Five-factor	170.202	80			

To further assess the potential influence of CMB, the ULMC approach was employed. Following prior research (Lian et al., 2018), two competing models were specified and compared. First, a baseline CFA model (M1) was estimated, in which all measurement items loaded only on their respective theoretical constructs. Subsequently, an alternative model (M2) was constructed by introducing a latent method factor, with all measurement items allowed to load simultaneously on both their corresponding trait factors and the common method factor.

The fit indices of the two models were then compared to evaluate the extent of common method variance. Specifically, if the changes in model fit indices between M1 and M2 (i.e., ΔRMSEA and ΔSRMR < 0.05; ΔGFI, ΔCFI, and ΔTLI < 0.10) remain within the recommended thresholds, this indicates that the inclusion of the method factor does not substantially improve model fit, suggesting that CMB is unlikely to pose a serious concern (Lian et al., 2018).

As shown in Table 4, the differences in fit indices between the two models are all within the acceptable ranges, indicating that the measurement model is stable and that CMB is not a significant threat in this study.

**Table 4:** ULMC test results.

Model	χ^2^	df	χ^2^/df	GFI	CFI	TLI	RMSEA	SRMR	ΔGFI	ΔCFI	ΔTLI	ΔRMSEA	ΔSRMR
M1	170.202	80	2.128	0.936	0.959	0.946	0.058	0.045	0.021	0.019	0.019	−0.011	−0.029
M2	112.5	65	1.731	0.957	0.978	0.965	0.047	0.016					

Δ values represent changes in fit indices for M2 relative to M1.

### Model measures assessment

As shown in Table 5, the measurement model demonstrates good reliability and convergent validity. All standardized factor loadings range from 0.634 to 0.858, exceeding the recommended threshold of 0.60. Cronbach's α values range from 0.762 to 0.867, indicating strong internal consistency across all constructs. Composite reliability (CR) values range from 0.763 to 0.867, all surpassing the recommended threshold of 0.70. The AVE values range from 0.518 to 0.684, exceeding the recommended threshold of 0.50. These results provide strong evidence of convergent validity (Fornell and Larcker, 1981; Chen et al., 2021a).

**Table 5:** Reliability and convergent validity.

Latent variable	Measurement variable	Factor loadings	Cronbach's α	CR	AVE
PU	PU3	0.807	0.762	0.776	0.539
	PU2	0.75			
	PU1	0.634			
PEU	PEU3	0.745	0.787	0.763	0.518
	PEU2	0.701			
	PEU1	0.713			
IQ	IQ3	0.825	0.867	0.867	0.684
	IQ2	0.833			
	IQ1	0.824			
CA	CA1	0.662	0.764	0.768	0.525
	CA2	0.768			
	CA3	0.74			
WTA	WTA1	0.858	0.836	0.841	0.638
	WTA2	0.784			
	WTA3	0.751			

PU, perceived usefulness; PEU, perceived ease of use; IQ, information quality; CA, crisis awareness; WTA, willingness to adopt.

Discriminant validity was assessed using the Fornell–Larcker criterion. As presented in Table 6, the square root of the AVE for each construct was greater than its correlations with other constructs, indicating that all constructs are empirically distinct from each other (Shrivastava, 2025; Liu and Cheng, 2026).

**Table 6:** Discriminant validity.

Constructs	WTA	CA	IQ	PEU	PU
WTA	**0.799**				
CA	0.434	**0.725**			
IQ	0.636	0.34	**0.827**		
PEU	0.591	0.52	0.634	**0.720**	
PU	0.638	0.583	0.633	0.548	**0.734**

The bold values in the diagonal row are the square roots of the average variance extracted for the constructs in the research model.

PU, perceived usefulness; PEU, perceived ease of use; IQ, information quality; CA, crisis awareness; WTA, willingness to adopt.

### Model fit

Prior to hypothesis testing, the overall fit of both the measurement and structural models was evaluated using AMOS 26.0. As presented in Table 7, all fit indices for the structural model exceeded the recommended thresholds (e.g., χ^2^/df = 2.128 < 3.0, CFI = 0.959 > 0.90, RMSEA = 0.058 < 0.08, RMR = 0.045). These results demonstrate an acceptable fit between the empirical data and the hypothesized structural model.

**Table 7:** Fit indices for structural models.

Model	χ^2^	χ^2^/df	AGFI	NFI	TLI	CFI	RMSEA	RMR
Structural model	170.202 (*p* < 0.001)	2.128	0.904	0.926	0.946	0.959	0.058	0.045
Recommended criteria	/	< 3.0	>0.90	>0.90	>0.90	>0.90	< 0.08	< 0.05

### Path analysis

Following confirmation of an acceptable model fit, SEM was conducted to test the hypothesized relationships. The results are presented in Table 8. PU has a significant positive effect on WTA (β = 0.552, *p* < 0.001), supporting H1. Similarly, IQ shows a significant positive effect on WTA (β = 0.285, *p* < 0.001), supporting H3. These findings indicate that both PU and IQ play important roles in shaping WTA. In contrast, PEU does not have a significant effect on WTA (β = −0.007, *p* = 0.948). Likewise, CA does not significantly influence WTA (β = 0.019, *p* = 0.792). Therefore, H2 and H4 are not supported. Overall, the results suggest that PU and IQ are key drivers of WTA, whereas PEU and CA do not exert significant direct effects in the structural model.

**Table 8:** Hypothesis test results of the model.

Hypothesized relationship	β	*B*	*SE*	*Z*	*p*
H1: PU → WTA	0.552	0.592	0.125	4.746	^***^
H2: PEU → WTA	−0.007	−0.008	0.116	−0.066	0.948
H3: IQ → WTA	0.285	0.265	0.072	3.705	^***^
H4: CA → WTA	0.019	0.02	0.075	0.264	0.792

^***^p < 0.001.

PU, perceived usefulness; PEU, perceived ease of use; IQ, information quality; CA, crisis awareness; WTA, willingness to adopt.

### Analysis of the moderating effect of crisis awareness

As shown in Table 9, PU has a significant positive effect on WTA (*B* = 0.512, *p* < 0.001). CA does not have a significant effect on WTA (*B* = 0.035, *p* = 0.493). The interaction between PU and CA is not significant (*B* = −0.041, *p* = 0.201, and 95% CI [−0.104, 0.022]). Thus, CA does not moderate the relationship between PU and WTA. H5a is not supported.

**Table 9:** Moderating effect of CA on PU and WTA.

Predictors	*B*	*SE*	*t*	*p*	95% CI (lower)	95% CI (upper)
PU	0.512	0.053	9.66	< 0.001	0.408	0.616
CA	0.035	0.051	0.686	0.493	−0.065	0.135
PU × CA	−0.041	0.032	−1.281	0.201	−0.104	0.022
*R* ^2^	0.312					
*F*	50.415^***^					

^***^p < 0.001.

PU, perceived usefulness; CA, crisis awareness; WTA, willingness to adopt.

As shown in Table 10, PEU does not have a significant effect on WTA (*B* = 0.143, *p* = 0.486). CA is also not significant (*B* = −0.027, *p* = 0.897). The interaction between PEU and CA is not significant (*B* = 0.064, *p* = 0.265, and 95% CI [−0.049, 0.178]). Thus, CA does not moderate the relationship between PEU and WTA. H5b is not supported.

**Table 10:** Moderating effect of CA on PEU and WTA.

Predictors	*B*	*SE*	*t*	*p*	95% CI (lower)	95% CI (upper)
PEU	0.143	0.205	0.698	0.486	−0.26	0.546
CA	−0.027	0.211	−0.13	0.897	−0.442	0.387
PEU × CA	0.064	0.058	1.117	0.265	−0.049	0.178
*R* ^2^	0.274					
*F*	24.833^***^					

^***^p < 0.001.

PEU, perceived ease of use; CA, crisis awareness; WTA, willingness to adopt.

The moderating effect of CA on the relationship between IQ and WTA is presented in Table 11. IQ has a significant positive effect on WTA (*B* = 0.473, *p* < 0.001). CA also shows a significant positive effect (*B* = 0.247, *p* < 0.001). Importantly, the interaction term (IQ × CA) is statistically significant and negative (*B* = −0.083, *p* = 0.007), and the 95% confidence interval does not include zero (−0.143, −0.023). These results indicate that CA significantly moderates the relationship between IQ and WTA. Therefore, H5c is supported. Specifically, the positive effect of IQ on WTA decreases as the level of CA increases. It is noteworthy that the significance of CA differs between the SEM results and the PROCESS analysis. While CA does not exhibit a significant direct effect in the SEM model, it appears significant in the regression-based moderation analysis. This discrepancy may be due to differences in model specification. SEM estimates relationships at the latent level, whereas the PROCESS analysis is based on observed variables and may capture additional variance related to interaction effects.

**Table 11:** Moderating effect of CA on IQ and WTA.

Predictors	*B*	*SE*	*t*	*p*	95% CI (lower)	95% CI (upper)
IQ	0.473	0.046	10.299	< 0.001	0.383	0.563
CA	0.247	0.046	5.345	< 0.001	0.156	0.339
IQ × CA	−0.083	0.031	−2.714	0.007	−0.143	−0.023
*R* ^2^	0.361					
*F*	62.266^***^					

^***^p < 0.001.

IQ, information quality; CA, crisis awareness; WTA, willingness to adopt.

B, unstandardized coefficient.

To further interpret the interaction effect, a simple slope analysis was conducted, as shown in Table 12. The results indicate that the effect of IQ on WTA is strongest at low levels of CA (−1 SD: *B* = 0.556, *p* < 0.001), moderate at mean levels (*B* = 0.473, *p* < 0.001), and weakest at high levels of CA (+1 SD: *B* = 0.390, *p* < 0.001). This pattern confirms that the positive effect of IQ on WTA decreases as CA increases, further supporting the moderating role of CA.

**Table 12:** Simple slope analysis for the interaction effect of IQ and CA on WTA.

Score for CA	Effect	*SE*	*t*	*p*	95% CI (lower)	95% CI (upper)
−1 *SD*	0.556	0.054	10.316	< 0.001	0.450	0.662
*M*	0.473	0.046	10.299	< 0.001	0.383	0.563
*+1 SD*	0.390	0.057	6.905	< 0.001	0.279	0.501

## Discussion

### Direct effects of TAM variables and CA

This study validates the extended TAM framework regarding GenAI adoption in higher education. The results reveal that PU and IQ are significant predictors of WTA, whereas PEU and CA lack significant direct effects. These findings suggest that college English teachers tend to evaluate technology rationally, prioritizing utility, and content reliability over interface usability or generalized professional anxiety.

The significant role of PU aligns with foundational TAM assumptions. As GenAI becomes increasingly integrated into language education, teachers are more likely to adopt technologies that offer clear pedagogical benefits, such as assisting in generating reading materials and grading essays (Zuo et al., 2025). Similarly, IQ emerges as a critical determinant. Because current GenAI models may generate inaccurate or contextually inappropriate content, teachers remain highly sensitive to information quality. Their adoption decisions depend on content reliability to maintain academic rigor and linguistic accuracy (Elsayed, 2024).

Conversely, the insignificant direct effect of PEU indicates a shift from conventional educational technologies. Modern GenAI platforms operate via natural language processing, largely eliminating software programming barriers. Since teachers interact with AI through conversational prompts, usability is no longer a primary obstacle. This corroborates recent findings that PEU operates indirectly or becomes insignificant in advanced AI contexts (Lin et al., 2025). Furthermore, although GenAI introduces professional challenges, CA lacks a significant direct effect on WTA. High CA weakens the influence of information quality on adoption, but does not alter the effects of PU or PEU.

### The moderating role of CA

A key contribution of this study is revealing the selective moderating mechanism of CA. The results demonstrate that high CA significantly weakens the positive relationship between IQ and WTA, while leaving the effects of PU and PEU unaffected.

TRT provides a theoretical explanation for this conditional effect (Staw et al., 1981). The theory suggests that high professional threat can constrain cognitive processing. When English teachers experience elevated competitive pressure, they may prioritize rapid adaptation over careful assessment. To mitigate perceived professional vulnerability, anxious educators might utilize GenAI primarily to demonstrate technological compliance, thereby reducing their sensitivity to actual output quality.

While evaluating PU represents a general assessment of efficiency, evaluating IQ requires sustained cognitive effort, such as verifying AI-generated vocabulary and pedagogical logic. CA appears to specifically disrupt this cognitively demanding process. Under psychological pressure, teachers may skip careful quality checks and rely on simple judgments instead. This moderating effect suggests that crisis-induced cognitive overload may undermine teachers' careful evaluation of AI. This finding challenges the assumption that psychological pressure uniformly alters technology acceptance processes, and instead demonstrates that its impact is selective, targeting cognitively demanding evaluations such as information quality.

### Theoretical implications

This study advances the existing literature in three ways. First, it extends the boundaries of TAM by introducing CA, demonstrating that the translation of cognitive beliefs into behavioral intention is conditional upon psychological stress levels. This approach integrates information systems literature with educational psychology. Second, the study contextualizes TRT within human-computer interaction. It illustrates how psychological pressure can constrain rational information processing during the adoption of emerging technologies at the individual level. Third, the findings identify a specific boundary condition in technology acceptance. Rather than uniformly inhibiting adoption, excessive professional anxiety selectively weakens cognitively demanding evaluations (i.e., the assessment of IQ). This provides a more nuanced theoretical understanding of human-AI interaction in competitive professional environments.

### Practical implications

The findings offer practical insights for higher education administrators. First, institutions should avoid crisis-driven technology mandates. Since excessive anxiety can impair critical evaluation, administrators should foster a supportive environment that emphasizes human-AI collaboration rather than technological replacement (Zuo et al., 2025). Second, universities should establish clear policy frameworks detailing acceptable GenAI integration boundaries. Reducing operational ambiguity can help alleviate chronic anxiety, allowing teachers to focus on pedagogical quality rather than compliance concerns. Third, professional development programs should be updated. Beyond basic prompt engineering, training should focus on algorithmic literacy. Providing teachers with strategies to identify AI hallucinations will enhance their ability to assess IQ effectively (Elsayed, 2024). Additionally, assisting English teachers in transitioning from traditional content deliverers to AI-assisted learning facilitators can address the root of their professional concerns and support sustainable technology integration (Lu et al., 2024).

## Conclusion

This study extends TAM by incorporating IQ and CA to examine college English teachers' WTA GenAI. Structural analyses reveal that while PU and IQ significantly drive adoption intention, PEU and CA exhibit no direct effects. This indicates a rational prioritization of utility and content reliability. Crucially, CA functions as a conditional moderator that significantly weakens the positive relationship between IQ and WTA. Drawing on TRT, this finding suggests that elevated psychological pressure can induce cognitive narrowing, leading anxious language teachers to prioritize rapid compliance over rigorous content evaluation.

It should be noted that the measurement of CA in this study focuses on perceived urgency and performance-related pressure. While this captures observable psychological responses, it may not fully encompass broader dimensions such as professional identity threat. Future research should utilize longitudinal designs to track adoption behavior over time and adopt mixed-methods approaches to further explore the psychological mechanisms underlying teachers' CA.

## Data Availability

The original contributions presented in the study are included in the article/supplementary material, further inquiries can be directed to the corresponding author.
